# Data mining reveals the diversity of prophage endolysins targeting pathogenic enterococci

**DOI:** 10.1099/jgv.0.002292

**Published:** 2026-07-17

**Authors:** Finn O'Dea, Andrew Kinsella, Brooks J Rady, Andrew D Millard, Joseph Brown, Graham P Stafford, Stéphane Mesnage

**Affiliations:** 1 Molecular Microbiology, School of Biosciences, University of Sheffield, Sheffield, UK; 2 The Florey Institute of Infection, University of Sheffield, Sheffield, UK; 3 Becky Mayer Centre for Phage Research, Division of Microbiology and Infection, University of Leicester, Leicester, UK.; 4 Aparon Ltd, Lincoln, UK; 5 School of Clinical Dentistry, 19 Claremont Crescent, University of Sheffield, Sheffield, UK

**Keywords:** endolysin, *Enterococcus cecorum*, *Enterococcus faecalis*, *Enterococcus faecium*, enzybiotic, prophage

## Abstract

Antimicrobial resistance poses a critical global health threat, with enterococci among the leading contributors due to their intrinsic and acquired resistance to antibiotics. Clinically relevant species, including *Enterococcus faecalis* and *Enterococcus faecium* as well as the emerging poultry pathogen *Enterococcus cecorum*, highlight the need for alternative therapeutics across human and agricultural settings. Bacteriophages and their derived enzymes, particularly endolysins, offer promising antibacterial strategies but challenges such as phage resistance and limited lysin diversity hinder their application. In this study, we performed a large-scale analysis of prophage-encoded endolysins across these three enterococcal opportunistic pathogens, characterizing over 48,000 sequences. We identified 33 distinct domain architectures combining diverse catalytic and cell wall-binding domains (CBDs) including novel putative CBDs. These findings expand the known diversity of enterococcal lysins and provide a comprehensive resource for the rational design of stable, recombinant ‘enzybiotics’ to combat multidrug-resistant enterococcal infections.

## Data Summary

All genomes analysed in this work are available through GenBank. The data mining strategy was carried out using open-access software available through GitHub as described in the Methods section. The raw output of the search and sequences obtained after each filtering step are provided in Table S1e. Modelling data related to Fig. 6 is available online (https://doi.org/10.5281/zenodo.20285178).

**Impact Statement**

Antimicrobial-resistant enterococci threaten therapeutic options in both medicine and agriculture. Yet, the therapeutic potential of bacteriophage-derived endolysins (enzybiotics) is limited by an incomplete understanding of their natural diversity. By analysing more than 48,000 prophage-encoded lysins from *Enterococcus faecalis, Enterococcus faecium* and *Enterococcus cecorum*, this study provides the most extensive characterization of enterococcal lysin architectures to date. The identification of 33 distinct domain organizations, including a previously unrecognized cell wall-binding domain, substantially broadens the known functional repertoire of these enzymes. This work fills a major knowledge gap and offers a foundational resource for engineering stable, targeted enzybiotics to combat multidrug-resistant enterococcal infections.

## Introduction

Antimicrobial resistance (AMR) is a growing global health crisis, projected to result in ~10–40 million deaths annually by 2050 if current trends continue [1]. Among the major contributors to the global AMR crisis are enterococci. Enterococcal infections are especially prevalent in healthcare settings and are associated with high morbidity and mortality due to their intrinsic resistance to multiple antibiotics and their capacity to acquire additional resistance genes [2,3]. Enterococci are opportunistic pathogens responsible for a broad range of infections, including bloodstream and urinary tract infections [4], endocarditis [5] and device-associated infections [6]. *Enterococcus faecalis* (*Efs*) and *Enterococcus faecium* (*Efm*) are the two species of greatest clinical relevance, with vancomycin-resistant *E. faecium* (VRE) being designated a high-priority pathogen by the World Health Organization [7,8]. This classification reflects both its rising clinical significance and the substantial challenges in treating it via traditional antimicrobial therapy.

Beyond their threat to human health, enterococci are increasingly recognized as pathogens in agricultural settings, particularly in poultry. Over the past decades, the species *Enterococcus cecorum* (*Ecm*) has emerged as a prominent poultry pathogen, accounting for up to 7% of infections in the broiler farms of some countries [9]. AMR has been cited as a significant contributor to the rise in these infections [10,12]. The emergence of antibiotic-resistant enterococci in both human and agricultural populations underscores the need for a novel class of therapeutics, particularly given the declining pace of antibiotic development for these pathogens. *Efs*, *Efm* and *Ecm* have diverged from each other by a long evolutionary history and have been assigned to three distinct, deeply branching clades within the genus *Enterococcus* (*Efs*: clade I, *Efm*: clade II, *Ecm*: clade IV) [13].

Bacteriophages (phages) have emerged as a promising alternative or adjunct to antibiotics [14] with multiple reports demonstrating successful phage therapy in clinical and compassionate-use settings [14,16]. However, bacterial resistance to phages can arise rapidly through a variety of mechanisms, including the modification of surface receptors, activation of restriction-modification systems and other phage defence strategies [17]. Such resistance, alongside regulatory hurdles, limits the long-term effectiveness of phage therapy when used alone.

A complementary approach that could help overcome phage resistance involves the use of enzymes that cleave bacterial peptidoglycans (PGs) (the essential component of the cell envelope), thereby inducing lysis. These enzymes called ‘enzybiotics’ were originally defined as antibacterial proteins with enzymatic activity [18]. Enzybiotics are most commonly phage endolysins that degrade the PG at the end of the infection cycle to release virions [19,20]. Endolysins are combining enzymatically active domains (EADs) and cell wall-binding domains (CBDs) and have emerged as promising antimicrobials [21]. Access to high-throughput strategies to build libraries of lysins combining EADs and CBDs has been described to engineer enzymes with therapeutic potential [22]. Directed evolution also revealed that activities can be optimized [23]. Phage tail-associated proteins also contain PG-hydrolytic domains [24], but their therapeutic potential has been minimally explored, with the notable exception of the *Efs* metallopeptidase EnpA [25,26]. Unlike whole phage, endolysins do not require adsorption to a specific receptor or intracellular replication to exert their bactericidal effect but instead cleave PG. The structure of this essential component of the cell wall is extremely conserved, thereby reducing the likelihood of resistance being developed [27]. Multiple studies have demonstrated the efficacy of endolysins against diverse enterococcal strains, including VRE, with reports of bacterial resistance remaining exceedingly rare [28,29]. Despite this promise, the translation of endolysins into clinical application has been limited by challenges in scaling up production, including poor protein solubility and stability, and a relatively limited pool of natural lysin candidates to draw from during therapeutic development [30,31]. Recent studies have investigated the diversity of tail-associated lysins [24] and endolysins encoded by 171 virulent phages targeting enterococci [32], providing a useful resource to explore the therapeutic activity of these enzymes.

Building upon this research, our work presents a large-scale analysis of prophage-encoded endolysins within *Efm, Efs* and *Ecm*. Using a high-throughput pipeline, we characterized over 48,000 endolysin sequences and identified 33 unique domain architectures, modularly combining diverse EADs and CBD domains. We selected distinct amino acid sequences for each domain architecture that could be used to produce recombinant enzymes with therapeutic potential. Our analysis revealed a diverse repertoire of enterococcal phage lysins spanning multiple enzymatic classes and associated with a wide range of CBDs, including new putative binding domains. This work establishes a foundational resource for the rational design of novel endolysin-based therapeutics aimed at combating multidrug-resistant enterococci in both clinical and agricultural settings.

## Methods

### Prophage prediction

Genomes were downloaded from NCBI using Biopython v1.81 Bio.Entrez module, based on a list of all genomes identified as *Enterococcus* (taxid: 1350) that were present on Genbank at time of download (December 2023). Any genomes with <90% completeness or >10% contamination according to CheckM v1.2.3 [33] using ‘lineage_wf’ were discarded, and GTDB-Tk v2.4.0 [34] using ‘classify_wf’ was used to determine bacterial species. Prophages were identified with PhageBoost v0.1.7 [35], then run through VIBRANT v1.2.1 [36] and geNomad v1.5.2 [37] to check, with only prophages confirmed by PhageBoost and either VIBRANT or geNomad being kept. If both VIBRANT and geNomad identified it as a prophage, the longer genome was kept (the VIBRANT prediction was kept if both were the same length). Prokka v1.14.6 was used to annotate genomes using the PHROGS database [38].

### Identification of prophage-encoded endolysins and selection of representative endolysins

PHROGS annotations were used to identify genes annotated as ‘endolysin’. ‘Hypothetical proteins’ encoded by genes one or two genes away from a holin were also selected as putative endolysins.

From the raw output of the search, identical sequences along with sequences less than 115 amino acids (deemed to not encode complete domains) were removed. Following this step, clustal Omega [39] was used to identify and remove sequences which displayed more than 95% sequence identity. Interpro [40] and the NCBI conserved domain database [41,42] were then used to identify domains and to remove any truncated proteins which only made up a portion of an intact EAD. AlphaFold [43] predictions were performed to detect putative binding domains not detected by Interpro searches. Putative binding domains were subsequently run through Foldseek [44] to identify the closest homologues. BioEdit was used to generate sequence identity matrices and to align those sequences.

### *In silico* docking of enterococcal muropeptides into putative binding domains

Muropeptides from *Efs* (gmgm-AQK[AA]AA ~gm-AQK[AA]AA ~g) and *Efm* (gmgm-AQK[D]AA ~gm-AQK[D]AA ~g) were docked into the putative CBDs ΒSD, C-terminal Domain 1 (CTD1), CTD2 and DUF5648 using Boltz v2.2.1 [45]. To differentiate between genuine and spurious docking results, dockings with several positive control domains (LysM, SH3) and negative control proteins (GFP, RNBR) were also predicted (see https://doi.org/10.5281/zenodo.20285178), ‘Binding Analysis/Pluto Notebook.jl’ or https://pluto.land/n/s8dxd3y8 for a complete list of amino acid sequences). SMILES structures for each muropeptide were computed using PGFinder [46] and manually unreduced (by default, all SMILES structures generated by PGFinder contain reduced terminal sugars). Each SMILES structure was then paired with an amino acid sequence in a YAML file and fed to ‘boltz predict’ alongside the following flags: ‘--use_msa_server --use_potentials --step_scale 1 --recycling_steps 10 --diffusion_samples 25’. The full set of input YAML files and Boltz outputs can be found online (https://doi.org/10.5281/zenodo.20285178) under ‘Full Boltz-2 Results/’.

The ‘ligand_iptm’ scores for each protein-muropeptide docking were extracted using a custom Pluto.jl notebook [47,48] and visualized as a box plot using Makie.jl [49]. The custom ‘Docking Consistency’ metric was calculated in two steps: first, ligand atoms contacting the protein (< 8 Å) in at least 13 of the 25 Boltz predictions were selected using BioStructures.jl [50], and ligand atoms consistently contacting at least 10 protein atoms were determined to be ‘docked atoms’ (this selects for binding pockets or grooves as opposed to tangential points of contact). The final ‘docking consistency score’ was then calculated as the number of docked atoms divided by their Root Mean Square Deviation (RMSD) when compared to the top-ranked Boltz model. The full Pluto.jl Notebook code can be found online (https://doi.org/10.5281/zenodo.20285178), ‘Binding Analysis/Pluto Notebook.jl’, or at https://pluto.land/n/s8dxd3y8. Finally, hydrogen bonding between the most confident model of beta-sandwich domain (BSD) and the *Efs* muropeptide was computed and visualized in ChimeraX [51].

## Results and discussion

### Identification of putative endolysins encoded by prophages in pathogenic enterococci

**Fig. 1.**
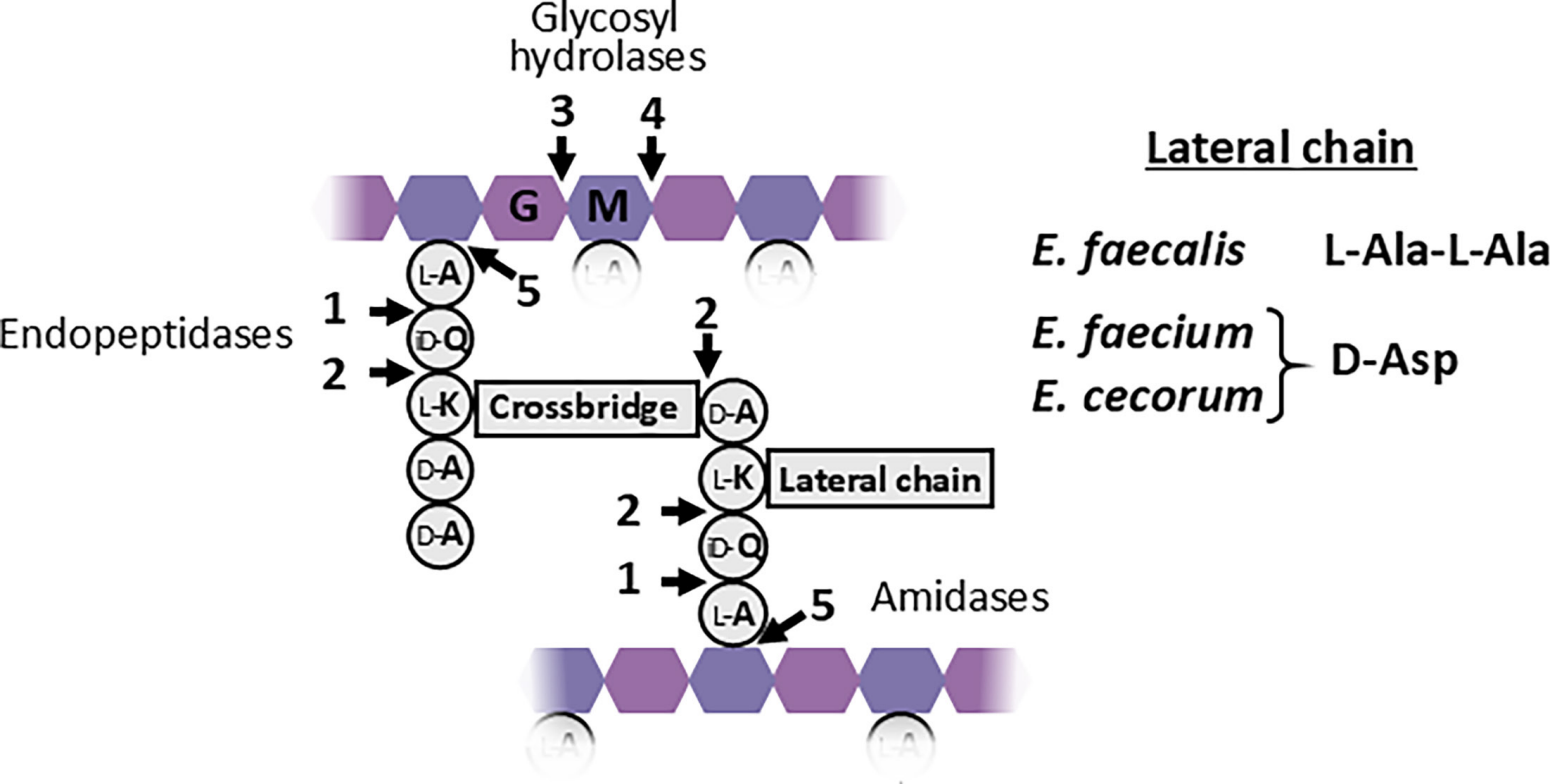
PG structure of selected enterococcal species and bonds cleaved by endolysins. Arrows indicate cleavage sites for different classes of enzymes. 1, l,d endopeptidases; 2, d,l endopeptidases; 3, *N*-acetylglucosaminidases (generate glycan chains with a GlcNAc at their reducing end); 4, *N*-acetylmuramidases (generate glycan chains with a MurNAc at their reducing end); 5, *N*-acetylmuramoyl-l-alanine amidases. No endolysin with a putative lytic transglycosylase EAD was identified in enterococcal prophages.

We sought to leverage the vast collection of available prophage genome sequences as an untapped resource for the discovery of novel enzybiotics. A total of 29,592 enterococcal genomes were analysed leading to the identification of 48,399 putative prophage endolysins across the three species chosen (Table S1, available in the online Supplementary Material): 299, 21,989 and 26,111 sequences for *Ecm*, *Efm* and *Efs*, respectively. After removing identical sequences and retaining only those longer than 115 residues (unlikely to contain any of the EAD of interest) ~10,000 candidate sequences remained. A final selection step retaining only endolysins with less than 95% sequence identity and those annotated to have enzymatic domains resulted in a refined dataset containing 245 endolysins across the three species (37 in *Ecm,* 82 in *Efm* and 126 in *Efs*; Table 1). Endolysins can be broadly classified into three groups based on the PG bond they cleave (Fig. 1). The first class corresponds to glycosyl hydrolases that target the glycan chain and includes *N*-acetylmuramidases and *N*-acetylglucosaminidases. Both cleave the β−1,4-glycosidic bonds between *N*-acetylmuramic acid (MurNAc) and *N*-acetylglucosamine (GlcNAc) residues, but muramidases generate a MurNAc reducing end, whereas glucosaminidases release a GlcNAc reducing end. No lytic transglycosylase was identified amongst enterococcal endolysin candidates. The second class corresponds to endopeptidases, which cleave peptide bonds either in the pentapeptide stem or in the lateral chain linking adjacent stem peptides. Endopeptidases can be stereospecific, cleaving either l,d or d,l bonds. The third class corresponds to *N*-acetylmuramoyl-l-alanine amidases (amidases), which cleave the bond between the first l-alanine in the peptide stem and MurNAc.

**Table 1.** Search strategy for endolysin identification. The output of each search step is provided in Table S1; all sequences resulting from the Interpro searches are also in Table S1

Search step	No. of sequence		
	*Ecm*	*Efm*	*Efs*
Raw output	299	21,989	26,111
Remove redundant sequences	138	4,177	6,283
Remove size (>115 residues)	106	3,845	6,103
clustal Omega (<95% identity)	65	289	373
Interpro (truncated domains)	37	82	126

EAD belonging to the three classes of endolysins described above were usually found associated with CBDs located at the C-terminus. A limited number of EADs were not associated with any CBD. Interestingly, some endolysins displayed a more complex domain organization, combining several EAD and/or CBDs.

### Endolysins with endopeptidase domains

**Fig. 2.**
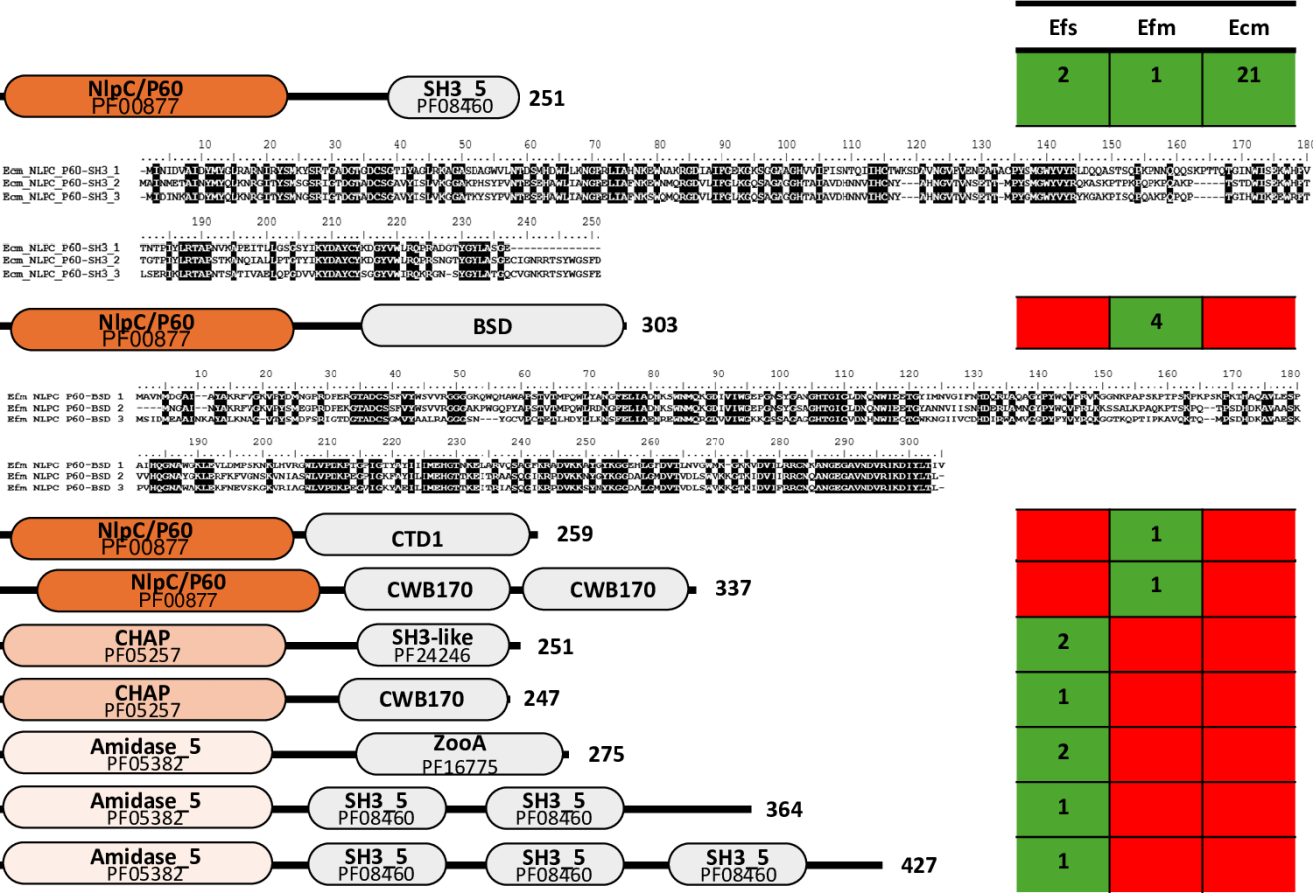
Domain architecture of endolysins containing a single endopeptidase EAD. Domain organizations of endopeptidases combined with various CBDs are shown. Boxes on the right-hand side show the number of sequence clusters (95% identity) in each species with a given domain architecture. An arbitrary cutoff of 75% identity led to the identification of three divergent groups of sequences for NlpC/P60-SH3 and NlpC/P60-BSD endolysins. Sequences are available in Table S1.

Analysis of enterococcal prophage genomes identified endolysins with four distinct endopeptidase domains: NlpC/P60 (PF0087), Amidase_5 (PF05382), M23 metallopeptidases (PF01551) and CHAP (cysteine, histidine-dependent amidohydrolases/peptidases; PF00527), the latter displaying either endopeptidase [52], amidase [53] or both activities [54]. M23 domains were only found in *Efm* prophage-encoded endolysins, whereas the three other EADs were present in all species studied. All these domains were found associated with other EADs (see later section), but NlpC/P60, CHAP and Amidase_5 domains were also found in endolysins with a single EAD (Fig. 2). Species-specific patterns of domain organization were found. Lysins encoded by *Efs* contained NlpC_P60, CHAP and Amidase_5 as standalone endopeptidase EADs, whereas those identified in *Efm* and *Ecm* harboured NlpC/P60 or Amidase_5 domains, suggesting a lineage-specific diversification of phage-encoded lytic systems. CHAP and NlpC/P60 endolysins are well characterized and exhibit potent antimicrobial activity against enterococci and other Gram-positive bacteria (see [28,53]). For example, the CHAP-domain-containing lysin LysEF-P10 displays strong lytic activity against *Efs* strains [55], and the NlpC/P60-containing LysPEF1-1 exhibited efficient bacteriolytic activity against multidrug-resistant *Efs* and *Efm* strains [56]. The Amidase_5 domain is most likely to display endopeptidase activity rather than amidase activity since it is structurally related to peptidases with a papain-like fold. No experimental evidence has shown the exact bond cleaved by the Amidase_5 EAD, but it has been suggested that it could have d-glutamine–l-lysine endopeptidase activity [57].

A total of nine distinct domain architectures combining a single endopeptidase EAD with one or two putative CBDs were identified. CBDs included SH3 (SRC homology 3) domains (PF08460 or PF24246), CWB170 domains (PDB 6L00) or ZooA (PF16775), described in the literature [58,61]. Two other uncharacterized putative CBD were identified and named the BSD based on its predicted structure and the CTD1. These domains were distributed differently between species and contributed to the observed diversity in lysin organization. CWB170, BSD or CTD1 were not detected by Interpro and were identified using AlphaFold and/or Foldseek. Structural prediction and alignment of the EADs revealed a high degree of conservation in overall fold architecture. Pairwise structural alignments of EADs (e.g. NlpC/P60 or CHAP) from distinct species yielded TM scores exceeding 0.7 across all comparisons, indicating significant structural similarity despite sequence divergence. Notably, EADs from *Efm* and *Ecm* displayed greater structural similarity to one another than to those from *Efs*, consistent with their similar PG composition. We further investigated amino acid sequence diversity within specific domain organizations. When a more stringent cutoff of 75% identity was set, we identified three divergent groups of sequences for two of the endopeptidase domain organizations described in Fig. 2 (NlpC/P60-SH3 and NlpC/P60-BSD) (Table S1).

### Endolysins with amidase domains

**Fig. 3.**
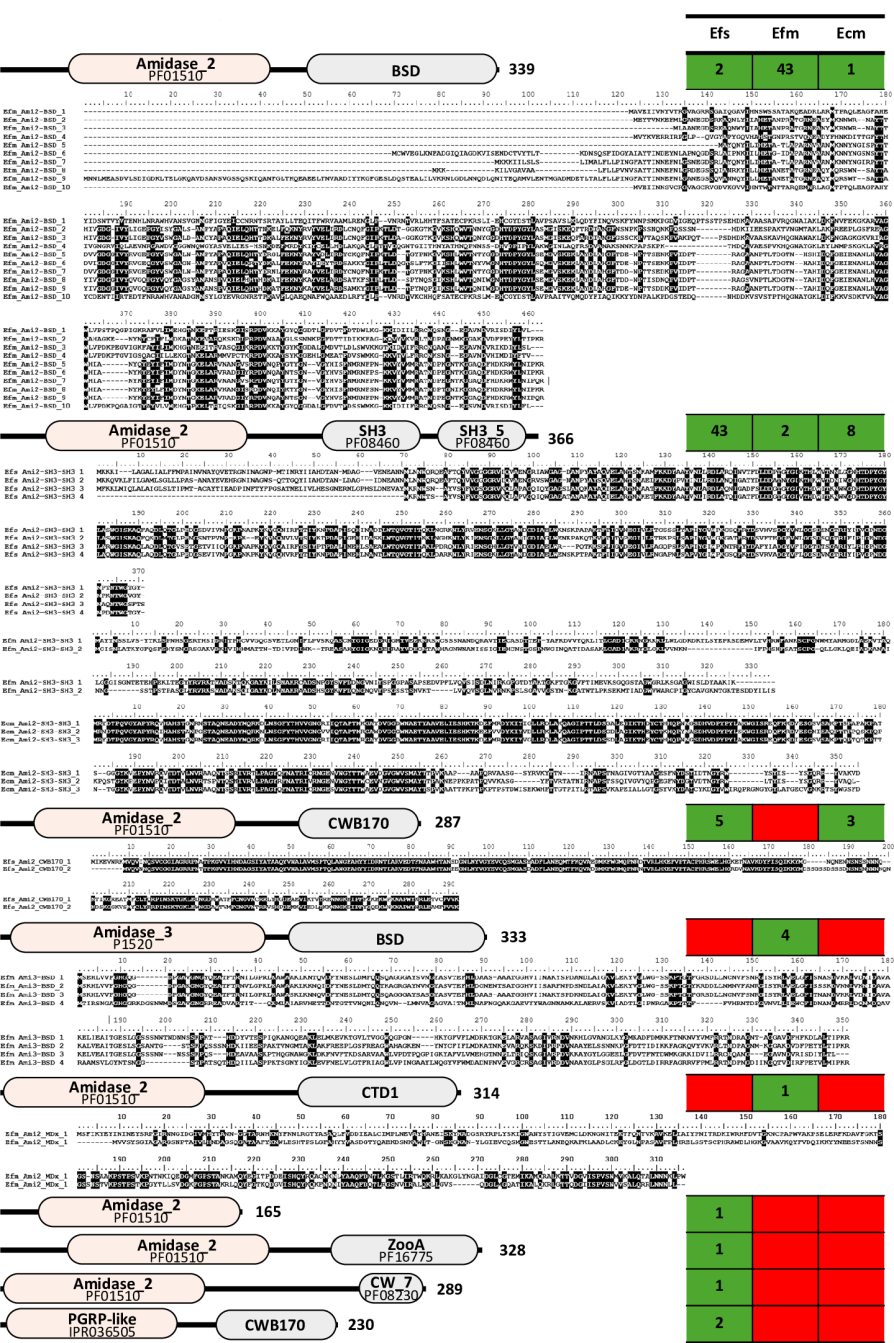
Domain architecture of endolysins containing an amidase EAD. Domain organizations of multiple amidase EADs combined with various CBDs are shown. Boxes on the right-hand side show the number of sequence clusters (95% identity) in each species with a given domain architecture. Further clustering at 75% identity led to the identification of divergent groups of sequences for several amidase endolysins. Sequences are available in Table S1.

Endolysins displaying distinct types of amidase EAD were identified (Fig. 3). These enzymes belonged to the Amidase_2 (PF01510) superfamily [which includes peptidoglycan recognition proteins (PGRPs) and CwlA-like autolysins], the Amidase_3 (PF01520) superfamily and one domain in the Amidase/PGRP superfamily (IPR036505). Amidase-containing lysins have been widely reported as effective antimicrobials against enterococci. Amidases such as PlyV12 or ORF9 exhibit broad lytic activity against vancomycin-resistant enterococci and display bactericidal activity against both *Efm* and *Efs* [62,63]. Only one amidase EAD was found in multi-catalytic enzymes; these EADs were mostly found in combination with a large variety of CBDs, including SH3, CWB170 and the previously described BSD and CTD1 domains, mirroring the modular architecture observed for endopeptidase lysins. Amidase endolysins were also found in association with ZooA-binding modules and CW_7 CBDs (PF08230 [64]), domains that were not observed in endopeptidase lysins. Additionally, more complex CBD arrangements were identified alongside the amidase domains, with multiple SH3 domains belonging to either the same or different classes being found at the C-terminus of amidase EADs. Species-specific patterns of catalytic domain distribution were again evident. Amidase_3 domains were detected exclusively in *Efm*, whereas Amidase_2 domains were distributed across all three species examined (*Efs*, *Efm* and *Ecm*). Two of the three CBDs identified in amidase-containing lysins (BSD and SH3) were present in all three species, while CWB170 was restricted to *Ecm* and *Efs*.

All pairwise structural comparisons of identical amidase EADs yielded TM scores exceeding 0.7, indicating a high degree of fold similarity across species. TM scores among amidase domains were more uniform than those observed for endopeptidase domains, likely reflecting the conserved biochemical target of these enzymes, which are all predicted to cleave the same MurNAc-l-Ala bond in PG. High sequence diversity was observed in amidase domain organizations. Four of the amidase-lysin domain architectures contained sequence clusters with less than 75% sequence identity. These included those encoding Ami2-BSD (ten *Efm* clusters), Ami2-SH3-SH3 (four *Efs* clusters; two *Efm* clusters; three *Ecm* clusters), Ami2-CWB170 (two *Efs* clusters) and Ami3-BSD (four *Efm* clusters) architectures. Representative cluster sequences are provided in Table S1.

### Endolysins with predicted muramidase domains

**Fig. 4.**
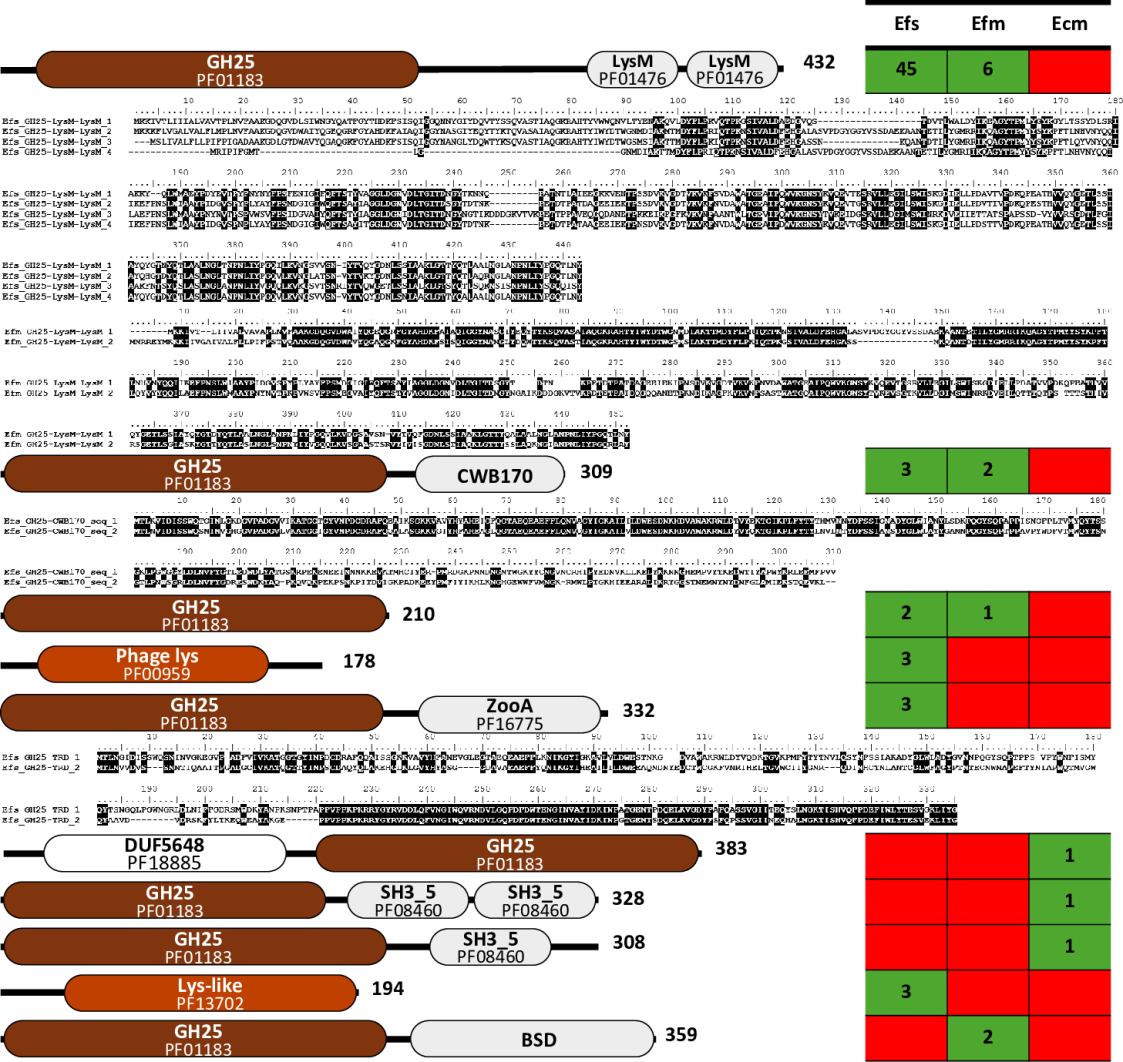
Domain architecture of endolysins containing a muramidase EAD. Domain organizations of multiple muramidase containing endolysins in combination with various CBDs. Boxes on the right-hand side show the number of sequence clusters (95% identity) in each species with a given domain architecture. Further clustering at 75% identity led to the identification of divergent groups of sequences for several muramidase endolysins. Sequences are available in Table S1.

A relatively large number of endolysins contained an EAD with muramidase activity. Three distinct EADs with this activity were identified: GH25 (PF01183), phage lysozyme (PF00959) and Lyz-like (PF13702), with GH25 being the most common (Fig. 4). Only one muramidase domain was found associated with another EAD, which contrasted with the endopeptidase domains which were often found in combination with other EADs. Few examples of endolysins with muramidase activity have been described in the literature, except for *Efs* AtlB, which contributes to septum cleavage and cell wall remodelling [65].

Muramidase EADs were observed in association with several CBDs: LysM (PF01476), SH3 (PF08240) and ZooA (PF16775), as well as the BSD and CW170 domains. Additionally, a single instance of a GH25 catalytic domain fused to a C-terminal DUF5648 domain was identified in *Ecm*. GH25 domains were only associated with SH3 CBDs in *Ecm*, in contrast to *Efm* and *Efs* which mostly contain LysM-associated GH25 domains. Domain architectures in *Efs* and *Efm* exhibited greater diversity, each incorporating three distinct identified CBD types: LysM, ZooA and SH3 (*Efs),* LysM, CWB170 and BSD (*Efm*). As previously described with the amidase and muramidase EADs, some complex endolysin did not contain any CBD, suggesting that these may be virion-associated PG hydrolases [20].

Structural comparisons of representative muramidase domains again demonstrated high fold conservation, with pairwise structural alignments yielding TM scores approaching 0.7, indicative of strong structural similarity despite sequence divergence. This structural conservation is consistent with the canonical lysozyme-like GH25 fold which comprises a predominantly α-helical architecture surrounding a conserved catalytic glutamate residue. Sequence diversity was again observed with three of the domain organizations including GH25-LysM-LysM (four *Efs* and two *Efm* clusters), GH25-CWB170 (two *Efs* clusters) and GH25-ZooA (two *Efs* clusters) (Table S1).

### Endolysins with multiple EADs

**Fig. 5.**
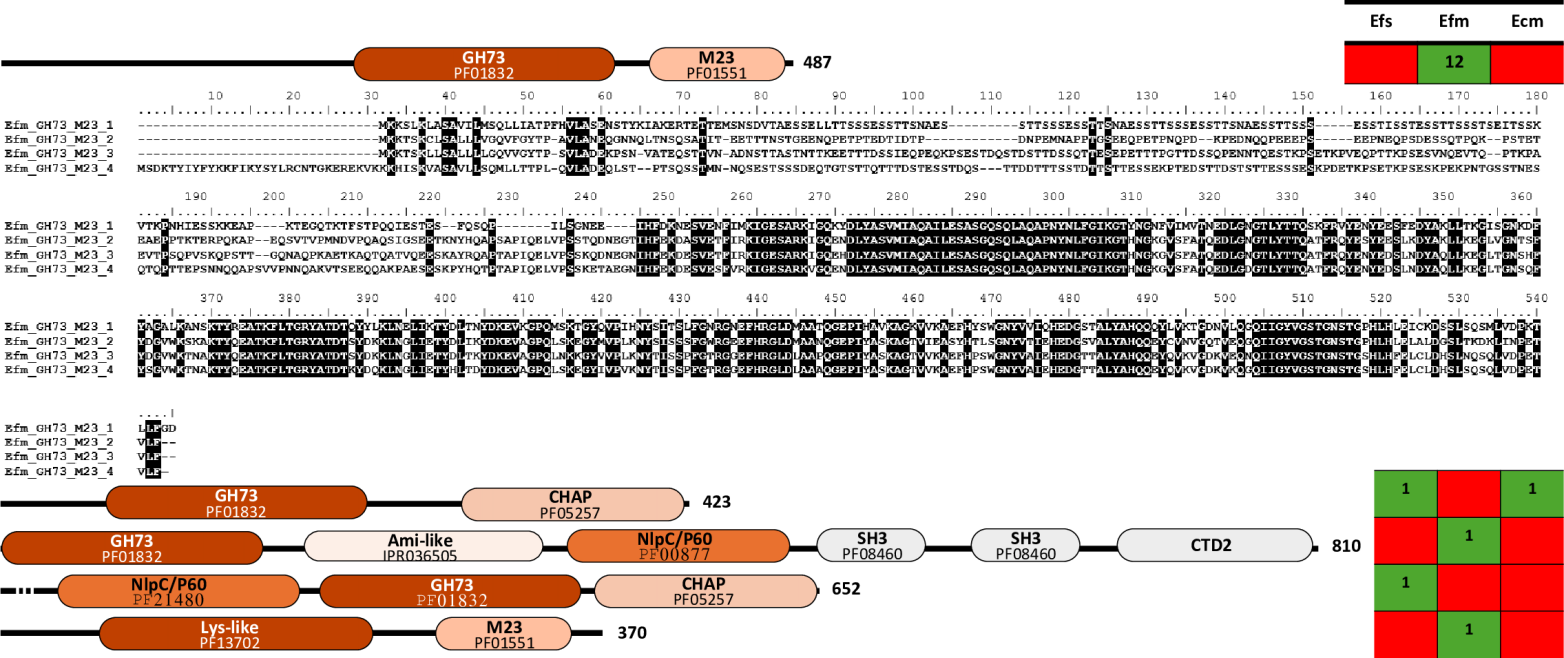
Domain architecture of endolysins with multiple EADs. Domain organizations of various endolysins with multiple EADs are shown. Boxes on the right-hand side show the number of sequence clusters (95% identity) in each species with a given domain architecture. An arbitrary cutoff of 75% identity led to the identification of divergent groups of sequences for several endolysins with multiple EADs. Sequences are available in Table S1.

All species studied encoded endolysins with multi-catalytic domain architectures. These included a glycosyl hydrolase domain (GH73 and lysozyme-like) and one or two peptidase domains (M23, NlpC/P60 or CHAP) and a single example of an amidase-like domain. Only one of these multi-catalytic lysins was found to also contain two SH3 CBDs as well as an unknown C-terminal domain that we called CTD2 (Fig. 5). This architectural complexity is consistent with prior observations that modularity enhances substrate recognition, catalytic efficiency and host specificity among Gram-positive-targeting lysins [66]. Interestingly, some of the EADs present in these multi-domain endolysins (GH73 and M23) were never detected as standalone EADs. The most common architecture was a GH73 fused to a C-terminal M23 EAD with no CBDs and was found exclusively in *Efm*. Other architectures were seen only once or twice. The 13 GH73-M23 lysins identified in *Efm* included five sequence clusters displaying less than 75% sequence similarity (Table S1), which mainly came from the predicted N-terminal intrinsically disordered region; limited sequence variability was also detected in both EADs.

### *In silico* characterization of putative novel cell wall-binding domains conserved across species

**Fig. 6.**
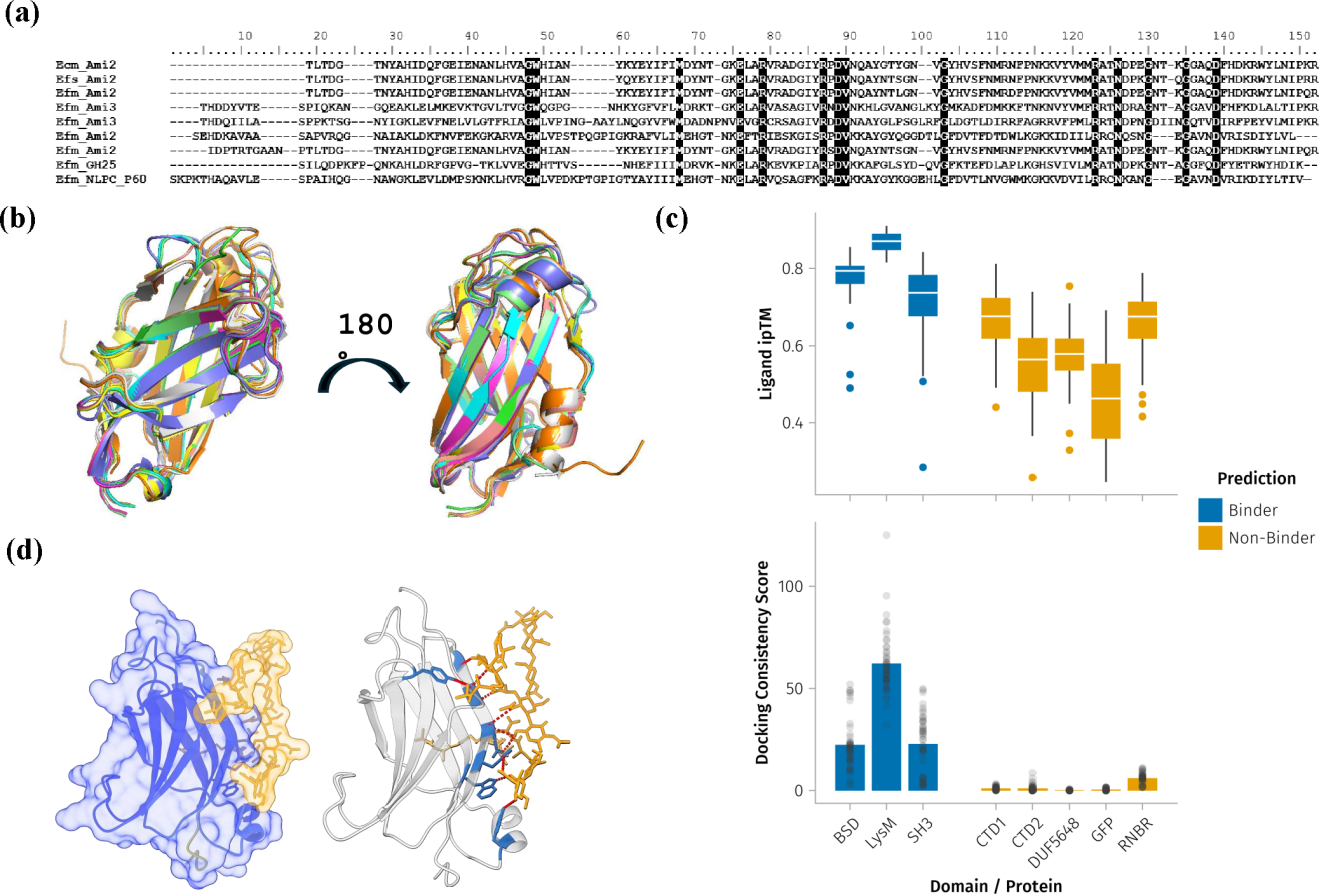
BSD domain sequence alignment, structural overlay, and muropeptide docking show BSD as a predicted binder with ligand ipTM and docking consistency scores comparable to known binders LysM and SH3. (a) Alignment of amino acid sequences encoding the BSD. (**b)** Structural alignment of AlphaFold predictions. (**c)** A box plot quantifying the ligand ipTM scores of 25 *Efm* and *Efs* muropeptide dockings for various domains/proteins, and the custom-calculated docking consistency scores for each of those 25 predictions. The results have been visually grouped into predicted binders and predicted non-binders by comparing ipTM and docking consistency scores to known binders (LysM, SH3) and random proteins (GFP, RNBR). (**d)** Docking of *Efs* muropeptide (gold) into the binding pocket of BSD (blue), including any predicted hydrogen bonding.

The endolysins identified in this study contained several well-characterized CBDs previously described in the literature [58,67]. In addition to these established domains, we identified three new domains that were present across the three species analysed and which, to our knowledge, have not been previously described. We have designated these novel modules BSD, CTD1 and CTD2. BSD is 135 amino acids in length (Fig. 6a), and structural predictions indicate that the domain adopts a compact fold consisting of two antiparallel β-sheets arranged in a sandwich-like configuration, with a single α-helix positioned at the C-terminal end of the domain (Fig. 6b). Despite limited sequence identity (Fig. 6a), the BSD fold is predicted to be highly conserved (Fig. 6b). CTD1 is 117 amino acids in length and is made up of mainly alpha helices. CTD2 on the other hand, is 122 amino acids in length and is composed of five antiparallel β-strands and two α-helices, one positioned in the middle of the structure and one at the C-terminal end of the protein. These structures seem to form stable scaffolds consistent with a potential role in cell wall recognition and binding.

Notably, BSD was identified in endolysins harbouring distinct EADs, including amidase (Ami_2/Ami_3), endopeptidase (NlpCP60) and muramidase (GH25) modules. This distribution across functionally different enzymatic classes suggests that the domain may act as a modular binding component capable of associating with diverse catalytic architectures. CTD1 was found in association with both amidase (Ami_2) and endopeptidase (NlpC_P60) domains, but CTD2 was only present in a single lysin, being found in a complex with three EADs and two additional CBDs (both SH3 domains).

To determine if these domains could be novel CBDs, muropeptide ligands from both *Efs* and *Efm* were docked into them *in silico*. For each docking, the ligand ipTM, a measure of docking confidence, was extracted and plotted in Fig. 6c (Top). Dockings with BSD showed similar ipTM scores to known CBDs (like LysM and SH3). CTD1 also showed high ipTM scores close to that of BSD indicating this too could be a cell-binding domain. CTD2, on the other hand, showed ipTM scores more like non-CBD controls (RNBR and GFP) suggesting it may not be involved in binding.

Despite displaying similar ipTM scores to known binders and BSD, the ipTM score of CTD1 was also close to that of barnase (RNBR, a ribonuclease included as a negative control). Visualization of the predicted structures revealed that, despite the high ipTM score, the docking position of muropeptides into CTD1 was inconsistent between models. To capture this ligand-pose inconsistency, we developed a custom ‘docking consistency’ score (see Methods). We found that this metric better captured the binding pocket and pose consistency indicative of a real ligand interaction and more cleanly separated our binder and non-binder controls. Docking consistency scores for our highest confidence binders (BSD, LysM and SH3 based on ipTM scores) were all high, indicating that the muropeptides were oriented consistently across the 25 models generated by Boltz (Fig. 6C, bottom). In contrast, RNBR and CTD1 showed low scores, indicating an inconsistency in muropeptide orientation across models. As a result of this inconsistency, we were unable to confidently predict CTD1 to be a cell binding domain.

Muropeptides from *Efs* (gmgm-AQK[AA]AA ~gm-AQK[AA]AA ~g) and *Efm* (gmgm-AQK[D]AA ~gm-AQK[D]AA ~g) fit neatly into defined grooves on the surface of the BSD (Fig. 6d; 3D models in https://doi.org/10.5281/zenodo.20285178 ‘BSD Visualizations/’) and formed ~10 hydrogen bonds to the protein. Interestingly, almost all those hydrogen bonds were to BSD’s peptide backbone, not to its amino acid sidechains. This may help to explain the relative lack of conserved amino acids in BSD sequences, as it suggests only the conserved backbone fold is required for muropeptide binding. Collectively, these findings support the classification of BSD as a structurally conserved and potentially functionally significant CBD in endolysins. It should be noted that, despite the low ipTM and docking consistency scores for both CTD1 and CTD2, their positioning relative to EADs (being found at the C-terminus) is similar to that of the other CBDs identified in this study. This suggests that the domains could still be involved in cell wall binding, although perhaps via non-PG component of the cell wall or untested PG substrate.

### Concluding remarks

This study significantly expands the known set of phage endolysins that could potentially be used as enzybiotics for the treatment of enterococcal infections. Data mining of prophage genomes revealed 33 unique architectures of enterococcal phage endolysins, approximately twice as many as previously reported from analyses of lytic phages. Our enumeration of the sequence diversity within each domain architecture serves as a valuable resource from which recombinant proteins can be expressed and evaluated as antimicrobials. Further experimental work is underway to characterize representative enzymes described in this study. The systematic expression of recombinant enzymes from identified groups and their comparative testing is required to confirm *in silico* predictions and determine therapeutic potential.

## Supplementary material

10.1099/jgv.0.002292 Table S1.

## Abbreviations

AMR: antimicrobial resistance
BSD: beta-sandwich domain
CBD: cell wall-binding domain
CHAP: cysteine, histidine-dependent amidohydrolases/peptidase
CTD: C-terminal domain
EAD: enzymatically active domain
*Ecm*: *Enterococcus cecorum*
*Efm*: *Enterococcus faecium*
*Efs*: *Enterococcus faecalis*
GlcNAc: *N*-acetylglucosamine
ipTM: Interface predicted TM-score
MurNAc: *N*-acetylmuramic acid
PG: peptidoglycan
PGRP: peptidoglycan recognition protein
RMSD: Root Mean Square Deviation
RNBR: *Bacillus amyloliquefaciens* barnase
VRE: vancomycin-resistant *Enterococccus faecium*
